# Cat scratch disease with generalized bone lesions in an immunocompetent child

**DOI:** 10.1080/22221751.2022.2127378

**Published:** 2022-10-26

**Authors:** Jing Wang, Wenjuan Chen, Meng Wang, Qiang Mi, Lijun Bo, Congli Yuan, Qing Cao

**Affiliations:** a Department of Infectious Diseases, Shanghai Children’s Medical Center, School of Medicine, Shanghai Jiaotong University, Shanghai, People’s Republic of China; b School of Agriculture and Biology, Shanghai Jiaotong University, Shanghai, People’s Republic of China; c Department of Hematology Oncology, Shanghai Children’s Medical Center, School of Medicine, Shanghai Jiaotong University, Shanghai, People’s Republic of China; d Shanghai Key Laboratory of Veterinary Biotechnology, Shanghai, People’s Republic of China

**Keywords:** *Bartonella henselae*, cat-scratch disease, bone comprise, malignancy, immunocompetent children

## Abstract

A 9-year-old immunocompetent girl with prolonged fever for 1 month was suspected of having a malignancy because of generalized bone abnormalities identified by MRI. Histopathology of liver tissues indicated the diagnosis of cat-scratch disease (CSD). Results of NGS, immunofluorescence and immunochemical assay confirmed the causative agent was *Bartonella henselae*. Paediatricians should increase their awareness of CSD as a cause for bone lesions, except for malignancy.

Cat-scratch disease (CSD) usually has a benign clinical course and recovers spontaneously within several weeks in immunocompetent children. Because of the limitation of clinical applicability of the *Bartonella* detection kit, many cases with disseminated *Bartonella* infection are easily misdiagnosed. Moreover, the direction of diagnosis may be misled by imaging examination, e.g. the children presenting with generalized bone lesions might be misdiagnosed as lymphoma or bone sarcoma. Here, we report a case of generalized visceral and bone lesions of CSD in an immunocompetent child who was suspected of having malignancy. She was diagnosed as CSD eventually by immunofluorescent and immunochemical assay, next-generation sequencing (NGS) and PCR detection of *Bartonella henselae* from biopsy tissues and blood. The purpose of this study is to introduce this rare manifestation of CSD and to improve the accuracy of understanding of CSD in immunocompetent children.

A nine-year-old girl was admitted because of fever of unknown origin for 1 month. She had a recurrent fever of 39–40°C 3–4 times per day. Except for febrile episodes, she did not report any other symptoms. Hepatosplenomegaly and a 20 mm*0.5 mm regional lymph node in her left neck were found on physical examination. Initial tests revealed mild anaemia (Hb 94 g/L), a mildly increased C-Reactive Protein (18 mg/L) and an increased erythrocyte sedimentation rate (90 mm/h) (The Reference Range:0–20 mm/h). The white blood cells, ferritin, LDH, immune function, and liver and kidney function were within normal limits. The blood and bone marrow cultures were negative for bacteria and fungi; virus serologies (rubella, CMV, EBV, HSV-1, and HSV-2) were negative, as were tests for tuberculosis (PPD, T-SPOT) and syphilis (VDRL, FTA-Abs). Tests for toxoplasmosis and other parasites (paragonimus, cysticercosis) were also negative. No autoimmune antibody (antinuclear antibody, double-stranded DNA antibody, antineutrophil cytoplasmic antibodies, extractable nuclear antigen antibodies, and rheumatoid factor) was identified. Genetic analysis by whole exon sequencing confirmed the patient was not immune deficient. She reported no history of consuming raw meat or unpasteurized dairy products. No family history of cancer or autoimmune disease was found. A cat and a dog were once kept in her house, and she was scratched on her right arm by the cat 6 months prior to admission. She was treated with intravenous ceftriaxone for 5 days and then meropenem and linezolid for 7 days in a local hospital. She still had a recurrent fever. At this point, she was suspected of having a malignancy because generalized bone abnormalities were observed on MRI scanning during hospitalization (Figure 1(A)). However, the results of bone marrow aspiration and biopsy failed to support any malignant diseases. The abdominal MRI scan showed irregular nodules on liver, spleen and kidneys (Figure 1(B)). Laparoscopic examination revealed many small white patchy nodules on the surface of the liver (Figure 1(C)).

**Figure 1.**
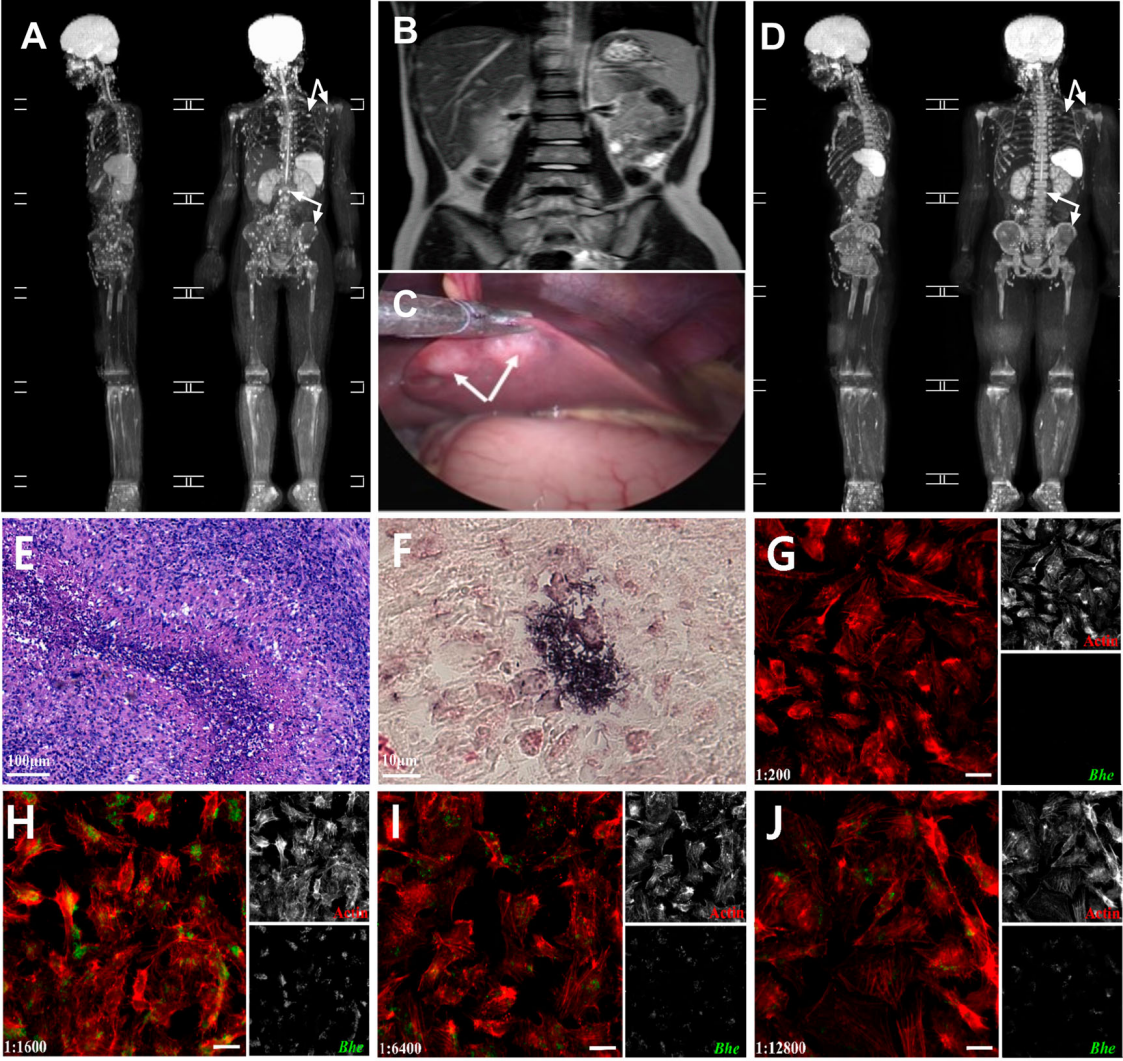
(A) MRI of the whole body showed multiple abnormal signals of bone (white arrow indicated). (B) Multiple lesions in liver, spleen and kidney on abdominal MRI-scan (white arrow on liver). (C) Laparoscopy identified multiple white nodules on the surface of liver (white arrow). (D) MRI of the whole body after 6 weeks of treatment with doxycycline. The abnormal signals improved significantly (white arrow). (E) Granulomatous inflammation, palisading histiocytes and abscess with necrosis (haematoxylin–eosin staining; original magnification, bar indicated 100 µm). (F) *Bartonella henselae* bacilli were detected in biopsied liver tissue by using mouse anti-*Bartonella henselae* polyclonal antibody (white arrow indicated). (G–J) Serodiagnosis demonstrating presence of *B. henselae* antibodies in serum from the patient using an immunofluorescence assay. G showed the serum collected from an age and sex matched health child (1:200 dilution); H–J showed the positive serum reaction of the patient from titre 1:1600–1:12,800. Bar in image G–J indicated a 10 μm.

Histopathology of the biopsied liver lesion demonstrated granulomatous inflammation and focal necrosis which raised suspicion for cat-scratch disease (CSD) (Figure 1(E)). Therefore, immunohistochemical staining of biopsied liver tissue was performed using a polyclonal mouse anti-*Bartonella henselae* antibody. Numerous small, rod-shaped bacteria were visualized in necrotic foci (Figure 1(F)). Immunofluorescence assay using Hela cells infected with intracellular *Bartonella henselae* ATCC 49882, was used for serodiagnosis. An extremely high titre to *Bartonella henselae* of 1:12,800 was obtained, which indicated active infection (Figure 1(G–I)). Affected liver and bone marrow samples were sent for NGS determination in order to exclude any other pathogens were involved. DNA and cDNA libraries were constructed, and were then loaded onto an Illumina Nextseq CN500 sequencer for 75 cycles of single-end sequencing. A total of 17 reads *Bartonella henselae* from the liver tissues were identified with a relative abundance of 1.9%. No pathogens other than *Bartonella henselae* were detected. Although the girl had generalized bone lesions, the *Bartonella* DNA was not detected in bone marrow. Subsequently, *Bartonella henselae* DNA was confirmed in liver tissue by PCR targeting on a 214-bp fragment of *gltA* gene (BHgf 5′- CGACTCTATTGATATTACAGATCCT-3′ and BHgb 5′-TCGAGTTAGCACCG GATT-3′). Affected liver and blood samples were submitted to cultivate *Bartonella henselae* on Columbia agar containing 5% defibrinated sheep blood (CBA) in a humidified atmosphere with 5% CO_2_ at 35°C. However, attempts to isolate *Bartonella* from liver samples had failed in a 30-day duration. Five days after the patient took the doxycycline, her temperature and C-reactive protein became normal. Follow-up 6 weeks with doxycycline after discharge showed that the visceral lesions and bone abnormalities had improved significantly (Figure 1(D)). The study was approved by the Institutional Review Board and the Ethics Committee of Shanghai Children’s Medical Center (SCMCIRB-K2022053-3).

When the fevers continued without a diagnosis, imaging examinations were performed. CT, PET-CT and MRI scans often have good sensitivity in leading to a diagnosis in patients with FUO [1]. In this patient, imaging scans showed hepatosplenic, kidney and disseminated bone lesions. Disseminated bone lesions can be observed in Langerhans cell histiocytosis, neuroblastoma, lymphoma, primary bone tumours and disseminated infections [2]. The hepatosplenic and kidney lesions could represent tumour invasion or infection. However, it was difficult to distinguish the potential causes of lesions from the radiological findings. Here, a biopsy of lesions in the liver was performed, which indicated a diagnosis of disseminated CSD along with the presence of anti-*B. henselae* antibodies. The history of a cat scratch provided further support for the diagnosis of disseminated CSD. Although bone lesion was found to be associated with CSD in immunocompetent children [3], it is not commonly observed in clinical patients. The CSD patient, in the present study, showed general bone lesions from spealbone, vertebral column to pelvic girdle, which is very unusual. Therefore, CSD should be included in the differential diagnosis in immunocompetent children with generalized bone lesions, especially in cases of FUO and with a history of cat contact or cat scratch.

Although isolation of *Bartonella* has been the gold standard for diagnosis of *Bartonella* infections, isolation of *B. henselae* from immunocompetent patients with CSD is highly challenged due to the very fastidious nature of *Bartonella* [4]. Therefore, biopsies with histopathological studies, including a Warthin-Starry silver stain, immunofluorescence tests for serum antibodies, and molecular detection are usually required for diagnosis [5].

The therapeutic approach to the treatment of *Bartonella* infections depends on the host’s immune status. Immunocompromised patients require prolonged treatment of 4–6 months, often with combined antimicrobial therapy, owing to the frequency of relapsed infection [6]. In immunocompetent children, the optimum duration of antibiotic therapy with disseminated *Bartonella* infection has not been determined [7]. Macrolides, tetracyclines, and rifampicin in monotherapy or combination are frequently used. Some studies suggested a total 6–12 weeks antibiotic course for vertebral or multifocal osteomyelitis CSD [8,9]. Some studies advised combination therapy should be given for at least 3 months’ duration in cases of severe, disseminated CSD. Relapses are more frequent if antibiotics are given for fewer than 3 months [10]. In our case, the therapeutic strategies of a 6-week course of tetracycline monotherapy were confirmed to be effective. The girl achieved clinical resolution in 6 weeks. Radiologic resolution of osteomyelitis was slower than the recovery of hepatosplenic granulomas. Because there are often other organs involved in disseminated CSD as in this case (liver, spleen, kidney or systemic bone lesions), it is reasonable to follow up with an MRI scan after several months to ensure a complete response to antimicrobial treatment.

Given that the manifestations of CSD are variable and easily confused with other infections and certain malignant diseases, a timely and accurate diagnosis is needed to avoid unnecessary treatments or interventions. *Bartonella* infection should be considered in addition to malignant disease when bone lesions are present in children.
